# Endometrial Assessment: When is it Necessary?

**DOI:** 10.4021/jocmr1684w

**Published:** 2013-12-13

**Authors:** Juliana Nicoletti Pessoa, Ana Carolina Lopes Freitas, Ronney Antonio Guimaraes, Jonnymar Lima, Helena Lucia Barroso dos Reis, Antonio Chambo Filho

**Affiliations:** a Gynecology and Obstetrics Department, Santa Casa de Misericordia School of Science, Brazil; b Gynecology and Obstetrics Department, Espirito Santo Federal University, Brazil

**Keywords:** Neoplasms, Endometrium, Age groups, Prevalence, Uterine hemorrhage, Risk factors, Carcinoma, Curettage, Ultrasonography, Endometrial hyperplasia

## Abstract

**Background:**

Endometrial cancer is the fourth most common cancer among women and the most common malignant neoplasm of the female genital tract in the USA. The onset is usually after the age of 50 and prognosis depends on the stage of disease at diagnosis. We aimed at determining the prevalence of high-risk endometrial lesions in women of different ages to establish a protocol for the indication of invasive diagnostic procedures.

**Methods:**

A retrospective study was conducted based on the descriptive and statistical analysis of histopathological records of 2,931 patients who underwent uterine curettage between January 2001 and December 2011 at our institution.

**Results:**

The risk of endometrial malignancy was about 10 times higher in patients aged 50 years or older than that in younger women. However, women with abnormal uterine bleeding had a higher prevalence of high-risk conditions, regardless of age.

**Conclusion:**

Atypical and complex endometrial hyperplasia and carcinoma can affect women of all ages, but are more common in patients 50 years of age or older. Thus, endometrial sampling is recommended as a routine procedure for all women 50 years of age or older with clinical indications of the disease and as a screening procedure for those undergoing hysterectomy.

## Introduction

Endometrial cancer is the fourth most common cancer among women and the most common malignant neoplasm of the female genital tract in the USA [1]. According to the Brazilian National Cancer Institute (INCA), the most common neoplasms among women in Brazil include non-melanoma skin tumors, tumors of the breast, cervix, colon and rectum, and lung and, less frequently, endometrial cancer [2]. Prognosis of endometrial cancer varies from excellent to very poor, depending on the stage of the disease at diagnosis [3]. The incidence of endometrial cancer has increased in the past decade although the incidence and mortality rates of other types of cancer have stabilized or even decreased. The increased incidence of endometrial cancer may be due to a rise in the diagnosis of advanced-stage cancers and in the incidence of histological subtypes of endometrial cancer with poor prognosis [1, 4].

Endometrial cancer affects predominantly women aged between 50 and 60 years, but 20-25% of cases are diagnosed in premenopausal women [5, 6]. The most common symptom in pre- and postmenopausal women is abnormal uterine bleeding, which is present in 90% of patients with endometrial cancer. Women with abnormal uterine bleeding should be tested because 10% of cases among postmenopausal women are caused by endometrial cancer [7]. Transvaginal sonography is an important tool for detecting endometrial disease in postmenopausal women with abnormal uterine bleeding, when the endometrial thickness is ≤ 4 mm [8, 9]. Few studies have performed routine endometrial sampling in asymptomatic women because of the invasiveness of the procedure. These studies have estimated prevalences of simple and complex hyperplasias ranging from 0.5 to 5% and prevalences of atypical hyperplasia and carcinoma to be < 1% [10, 11]. The American Cancer Society does not recommend routine screening for endometrial cancer in asymptomatic women [8], and the Canadian Cancer Society reported that routine transvaginal sonography or endometrial sampling does not result in lower mortality rates of endometrial cancer [8].

According to the terminology adopted by the World Health Organization (WHO), the severity of endometrial hyperplasia is classified as simple or complex based on architectural features, and as typical or atypical based on cytologic features, as initially described by Kurman et al [12]. Atypical hyperplasia is considered a precursor lesion, which may progress to endometrial cancer in 5% to 25% of patients [13, 14]. Some studies have reported that atypical hyperplasia may coexist with endometrial cancer. Histopathological examination of hysterectomy specimens revealed that 20% to 43% of patients previously diagnosed with endometrial atypical hyperplasia by biopsy also had endometrial cancer [13-16].

About 80% of endometrial cancers are histologically classified as type I endometrioid carcinomas with minimal myometrial invasion, arising from atypical complex hyperplasia, and especially affecting pre- and perimenopausal women. Type I endometrioid carcinomas are usually estrogen-receptor-positive tumors associated with hyperestrogenism and prior estrogenic stimulation. Several statistical studies have indicated a positive association of endometrial hyperplasia with obesity, diabetes, high blood pressure, breast cancer, chronic anovulation, estrogen-producing ovarian tumors, and genetic predisposition [10, 11, 14]. In contrast, type II endometrial cancers occur mostly in elderly or postmenopausal non-obese women, are more aggressive, have poor prognosis, and are frequently not associated with high estrogen levels [7, 14]. Because endometrial hyperplasia precedes the development of endometrial cancer by up to 10 years, the early diagnosis and treatment of this condition could significantly reduce the number of new cases of endometrial cancer [17].

Given the importance of the topic, the aim of this study was to estimate the current prevalence of high-risk endometrial lesions in women of different ages in the study institution to determine the cut-off age for indication of endometrial sampling as a screening procedure for endometrial cancer. This information makes it possible to focus on the endometrial screening of patients in high-risk groups and decrease the number of invasive procedures in low-risk groups, reducing health care costs and the risk of morbidity associated with invasive diagnostic procedures.

## Materials and Methods

The study was approved by the Research Ethics Committee of the Santa Casa de Misericordia School of Science of Vitoria, Brazil (approval number 039/2011) and was conducted in full accordance with ethical principles, including the World Medical Association Declaration of Helsinki (59th WMA General Assembly, Seoul, October 2008) [18]. Patient anonymity was ensured.

This was a retrospective study based on the descriptive and statistical analysis of data obtained from medical records of patients who were treated between January 2001 and December 2011 at the Obstetrics and Gynecology and Pathology Services of the Santa Casa de Misericordia School of Science Hospital.

The medical records of 2,931 patients who underwent uterine curettage were reviewed and the parameters: histopathological findings, age, and clinical indications were recorded. Thirty-eight of these patients were excluded for incomplete records, leaving 2,893 cases for the analysis. All endometrial curettage samples were collected at the obstetrics and gynecology service by different examiners and analyzed by clinical pathologists of the pathology service of the same institution.

The histopathological findings were classified according to the WHO classification as simple or complex hyperplasia, typical or atypical hyperplasia, and endometrial cancer, including all histological subtypes [12].

The subjects were stratified by age (≤ 29 years; 30 - 49 years; and ≥ 50 years), clinical indications, and presence or absence of uterine bleeding.

Statistical analysis was carried out using the Statistical Package for the Social Sciences (SPSS) 11.5 for Windows (SPSS Inc., Chicago, IL, USA). Risk of endometrial malignancy was estimated using odds ratio (OR) with a 95% confidence interval (CI). The chi-square test was used to test for association between uterine bleeding (presence or absence) and histopathological findings (benign or malignant endometrial disease). All statistical tests were performed at a significance level of 0.05 (P < 0.05).

## Results

The clinical indications among the 2,893 patients who underwent uterine curettage were uterine bleeding alone (n = 795), and uterine bleeding combined with polyps (n = 747), myoma (n = 323), endometrial thickening (n = 695), or other minor problems. Abnormal uterine bleeding was present in 36.39% of patients, while 63.61% of patients had no pathological bleeding.

Histopathological examination revealed 2,779 benign endometrial lesions, of which the most common were endometrial atrophy, polyps, and simple endometrial hyperplasia without atypia, as well as other benign changes including dysfunctional and proliferative endometrium. One hundred and fourteen histopathological findings were suspicious for malignancy, 13 were simple endometrial hyperplasias with atypia, 19 were complex hyperplasias, and 82 were endometrial cancers including the different histological subtypes (Table 1).

**Table 1:** Histopathological Findings

Histopathological findings	N	%
Insufficient material	195	6.74
Endometrial atrophy	102	3.53
Polyps	980	33.87
Other benign changes	1,302	45.00
Simple hyperplasias without atypia	200	6.91
Simple hyperplasias with atypia	13	0.45
Complex hyperplasias	19	0.66
Endometrial cancer	82	2.83
Total	2,893	100

For patients with abnormal uterine bleeding, 983 histopathological findings were benign and 69 were malignant, while for patients who had no bleeding, 1,796 findings were benign and 45 were malignant (Table 2). There was a significant association of uterine bleeding with the presence of endometrial malignancy (P < 0.05).

**Table 2:** Correlation Between Endometrial Malignancy and the Presence or Absence of Uterine Bleeding

Uterine bleeding	Benign lesion	%	Malignant lesion	%	Total
Present	983	93.44	69	6.56	1,052
Absent	1,796	97.55	45	2.45	1,841
Total	2,779	96.06	114	3.94	2,893

According to age group, it was found that 122 of 124 histopathological findings were benign and only 2 were malignant for patients aged ≤ 29 years; 1,755 findings were benign and 16 malignant for patients 30 - 49 years old, and 902 findings were benign and 96 malignant for patients aged ≥ 50 years (Table 3).

**Table 3:** Uterine Malignancy by Age Group

Age group (years)	Benign lesion	%	Malignant lesion	%	Total
≤ 29	122	98.39	2	1.61	124
30 - 49	1,755	99.10	16	0.90	1,771
≥ 50	902	90.38	96	9.62	998
Total	2,779	96.06	114	3.94	2,893

Patients aged ≥ 50 years had a higher risk of endometrial malignancy than patients aged ≤ 29 years (OR = 6.49; 95% CI = 1.55 - 38.54; P < 0.01) and those aged 30 - 49 years (OR = 11.67; 95% CI = 6.67 - 20.71; P < 0.01). The relative risk of endometrial malignancy for patients ≥ 50 years of age was 1.09 when compared with those ≤ 29 years of age and 1.10 when compared with those aged 30 - 49 years.

## Discussion

Endometrial cancer is the fourth most common cancer in women in the USA. It is estimated that 34,000 new cases of endometrial cancer were diagnosed and 6,000 deaths were caused by endometrial cancer in 1996 in the USA, with incidences ranging from 12/100,000/year in women aged ≤ 40 years to 100/100,000/year in women aged ≥ 60 years [19]. In Brazil, it is estimated that 5,685 new cases of endometrial adenocarcinoma occur annually, with a mean incidence of 7.6/100,000/year, ranging from 2/100,000/year in the Northern Region of the country to 9.9/100,000/year in the Southwest Region [2].

Our results revealed an overall incidence of endometrial cancer of 2.83%, which rises to about 4% if precursor lesions for malignancy are included. These estimated incidences are much higher than those reported for the general population, because in this study we evaluated data from a specific population seen at a tertiary hospital and who were presenting symptoms of endometrial cancer or clinical indications for endometrial screening. However, our results by age group are consistent with those reported for the general population, in which the incidence of endometrial malignancy increases for patients aged ≥ 50 years (Table 3).

We observed that patients aged ≥ 50 years had a higher risk of endometrial malignancy than patients aged ≤ 29 years (OR = 6.49; 95% CI = 1.55 - 38.54; P < 0.01) and those aged 30 - 49 years (OR = 11.67; 95% CI = 6.67 - 20.71; P < 0.01). The relative risk of endometrial malignancy for patients aged ≥ 50 years was 1.09 when compared with those aged ≤ 29 years and 1.10 when compared with those aged 30 - 49 years. This shows that the risk of endometrial cancer in women 50 years of age or older is about 10 times higher than that in younger women and indicates the importance of endometrial sampling for this age group.

Iram et al [20] conducted a study with 3,006 premenopausal women with abnormal uterine bleeding and reported prevalences of hyperplasia and carcinoma significantly higher in women aged 45 - 50 years than in younger women. The authors suggested a cut-off age of 45 years for endometrial sampling in patients with abnormal uterine bleeding. Our results revealed prevalences of malignant endometrial disease of 6.56% in patients with uterine bleeding, and of 2.45% in patients who had no uterine bleeding, with a significant association of uterine bleeding with the presence of endometrial malignancy (P < 0.05). Irregular menstrual bleeding has always been considered a warning sign and a high-risk factor for malignancy. Crissman et al [17] carried out a study with 54 endometrial cancer patients aged ≤ 40 years, and reported that 81% of patients presented with irregular menstrual bleeding [20]. Similar findings have been reported more recently by Iram et al [20], who observed that 22 of 23 endometrial cancer patients aged ≤ 45 years had irregular menstrual bleeding.

Atypical or complex endometrial hyperplasia and endometrial cancer can affect women in any age group, but are more common among those aged 50 years or older. Based on our results, we recommend endometrial sampling for all women aged ≥ 50 years who have clinical indications of endometrial malignancy, and, occasionally, for those undergoing hysterectomy regardless of whether they are pre- or postmenopausal. On the other hand, invasive diagnostic procedures are not indicated for asymptomatic women younger than 50 years because patients at this age group were found to be at statistically low-risk for endometrial cancer development. Endometrial sampling in younger women increases health care costs and the risk of morbidity associated with invasive diagnostic procedures. However, premenopausal women with irregular menstrual bleeding should also undergo endometrial sampling, because this symptom is highly associated with endometrial malignancy.

The data analyzed in this retrospective study was extracted from hospital records; hence, it was impossible to provide information on underlying diseases.
